# Innovative Strategies for Expanding Dementia Education: Leveraging Grant Funding and Partnerships for Engagement

**DOI:** 10.1093/geroni/igaf122.415

**Published:** 2025-12-31

**Authors:** Ashley Haas, Jessica Bibbo, Megan Huth

**Affiliations:** Benjamin Rose Institute on Aging, Cleveland, Ohio, United States; Benjamin Rose Institute on Aging, Cleveland, Ohio, United States; Benjamin Rose Institute on Aging, Cleveland, Ohio, United States

## Abstract

This session will present how the organizational grant partners successfully leveraged an Administration for Community Living (ACL) Alzheimer’s Disease Programs Initiative (ADPI) grant to expand dementia education to the public. The three-year grant focused on serving urban and rural communities within the state of Ohio. A dementia education webinar series was developed to engage community members. The series delivered 24 sessions, reaching over 2,782 community members, including professionals, caregivers, and individuals living with dementia. The project saw substantial growth each year, marked by increases in both registration and attendance. The average number of registrants rose from M = 141 (SD = 40.07) in Year 1 to M = 235 (SD = 89.68) in Year 3, while average attendance increased from M = 84 (SD = 34.36) in Year 1 to M = 130 (SD = 59.97) in Year 3. Initially focused on a specific geographic area in Ohio, the project expanded its reach nationwide and internationally by employing innovative engagement strategies, reaching participants from all 50 states, Washington, DC, and five additional countries. The national reach grew significantly each year, with an average increase in national participants from M = 41 (SD = 17.21) in Year 1 to M = 85 (SD = 45.02) in Year 3. This session will highlight how the strategic use of grant funding and collaborative partnerships can enhance outreach and foster impactful engagement.

